# Effect of oral administration of low-dose follicle stimulating hormone on hyperandrogenized mice as a model of polycystic ovary syndrome

**DOI:** 10.1186/s13048-015-0192-9

**Published:** 2015-10-06

**Authors:** Irene Tessaro, Silvia C. Modina, Federica Franciosi, Giulia Sivelli, Laura Terzaghi, Valentina Lodde, Alberto M. Luciano

**Affiliations:** Reproductive and Developmental Biology Laboratory, Department of Health, Animal Science and Food Safety, Università degli Studi di Milano, Via Celoria 10, Milan, 20133 Italy; Interdepartmental Research Centre for the Study of Biological Effects of Nano-concentrations (CREBION), Università degli Studi di Milano, Via Celoria 10, Milan, 20133 Italy

**Keywords:** Polycystic ovary syndrome, Ovary, Follicle cyst, Mouse, Oral administration, Bioactive peptides, Gonadotropins, Animal model

## Abstract

**Background:**

Polycystic Ovary Syndrome (PCOS) is a widespread reproductive disorder characterized by a disruption of follicular growth and anovulatory infertility. In women with PCOS, follicular growth and ovulation can be induced by subcutaneous injections of low doses of follicle stimulating hormone (FSH). The aim of this study was to determine the effect of oral administration of recombinant human FSH (rhFSH) on follicle development in a PCOS murine model. Moreover, since it is unlikely that intact rhFSH is present into the circulation after oral administration, the biological activity of a peptide fragment, derived from the predicted enzymatic cleavage sites with the FSH molecule, was investigated *in vitro* on cumulus-enclosed oocytes (COCs).

**Methods:**

Female peripubertal mice were injected with dehydroepiandrosterone (DHEA) diluted in sesame oil for 20 consecutive days and orally treated with a saline solution of rhFSH. A control group received only sesame oil and saline solution. At the end of treatments, blood was analyzed for hormone concentrations and ovaries were processed for morphological analysis. The presumptive bioactive peptide was added during *in vitro* maturation of bovine COCs and the effects on cumulus expansion and on maturation rate were evaluated.

**Results:**

DHEA treatment increased serum levels of testosterone, estradiol and progesterone as well as the percentage of cystic follicles. Orally administered rhFSH restored estradiol level and reduced the percentage of cystic follicles. Despite these results indicating a reduction of the severity of PCOS in the mouse model, the presumptive bioactive peptide did not mimic the effect of rhFSH and failed to induce bovine cumulus expansion and oocyte maturation *in vitro*.

**Conclusions:**

Although further studies are needed, the present data supports the concept that orally administrated FSH could attenuate some of the characteristic of PCOS in the mouse model.

**Electronic supplementary material:**

The online version of this article (doi:10.1186/s13048-015-0192-9) contains supplementary material, which is available to authorized users.

## Background

Polycystic Ovary Syndrome (PCOS) is a widespread reproductive and endocrinologic disorder, which accounts for approximately 80 % of women with anovulatory infertility [1, 2]. PCOS is characterized by hyperandrogenism and polycystic ovaries, in addition to anovulation [3]. This syndrome can also be associated with metabolic issues including obesity, insulin resistance, hyperinsulinemia, and type 2 diabetes mellitus, besides cardiovascular problems, breast and endometrial cancers, and neurological and psychological effects on quality of life [4, 5].

In affected women, the normal ovarian function is disturbed mostly by hyperandrogenism and by the elevated serum concentrations of luteinizing hormone (LH, [6, 7]), thus resulting in multiple small cysts [8, 9]. A nearly universal finding in PCOS is an increased gonadotropin-releasing hormone (GnRH) pulse frequency, which favors LH production over follicle stimulating hormone (FSH) [10, 11]. The increased LH subsequently promotes theca cell production of androgens, while the relative FSH deficiency reduces the ability of granulosa cells to convert androgen into estrogen and impairs follicle maturation and ovulation [12].

Ovulation induction protocols can be used to restore fertility in PCOS patients [13]. One protocol involves ovarian stimulation by subcutaneous FSH injection [13, 14]. However, because of the large number of small antral follicles that are sensitive to FSH [15], women with PCOS have a higher risk in developing ovarian hyper-stimulation syndrome (OHSS) in response to FSH treatment [16]. To reduce this risk, low-dose administrations of injectable FSH have been used [17, 18]. For this purpose, the most appropriate regime is the step-up protocol, in which the FSH dose is gradually increased until follicular development is observed, and then maintained until follicular selection is achieved [19, 20].

Regardless of good pregnancy and live birth rates [17], ovarian stimulation with FSH is actually considered a second-line treatment for the PCOS patients with infertility [13]. This is mainly due to the lack of an oral formulation, the elevated price, and the potentially severe adverse effects, such as multiple pregnancy and OHSS (reviewed in [13]). The basic concern about oral formulation is the low bioavailability of FSH that stem from stomach enzymatic degradation and poor penetration of FSH peptides across the intestinal membrane [21, 22]. As a consequence it is clear that the research leading to improved oral FSH therapy could ultimately lead to the development of innovative and more comfortable treatments for PCOS.

Androgen-treated rodents have been widely used as models to study both reproductive and metabolic deficits of PCOS (reviewed in [23–25]). These studies demonstrate that dihydroepiandrosterone (DHEA) is able to induce many of the salient features of the human PCOS condition, such as hyperandrogenism, insulin resistance, altered steroidogenesis, acyclicity, abnormal maturation of ovarian follicles, and anovulation in rodents models [26–29]. Then the present work aims to assess first of all, the effect of oral administration of low-dose FSH on the morphological and endocrine function of the ovaries of hyperandrogenized mice. Subsequently, since intact FSH is unlikely to be transferred from the gastrointestinal tract into the circulation, the effect of a minimal amino acid sequence, derived from the analysis of the enzymatic cleavage sites, was analyzed on an *in vitro* system of bovine oocyte maturation (IVM). Recombinant human FSH is commonly used in *in vitro* maturation to stimulate meiotic resumption and cumulus expansion both for research purpose and for application in assisted reproductive technologies in bovine *in vitro* embryo production [30–34] and its effectiveness is sustained by a growing body of literature. Moreover, the bovine IVM model represents a highly standardized protocol [35, 36], particularly efficient and versatile, which allows at the same time to limit the use and sacrifice of experimental animals.

## Methods

All procedures were carried out in accredited animal care facilities at the University of Milan, maintained by the Center for Laboratory Animal Care. The experimental protocol was approved by the University of Milan Ethics Committee and by the Responsible for Laboratory Animal Care veterinarian and in accordance with National (Italian DLT 27/01/1992 n. 116) and European (European Directive 86/609/EEC on Animal Care and use for scientific and other experimental purposes) legislation.

The chemicals used in this study were purchased from Sigma Chemical Company (St. Louis, MO, USA) except for those specifically mentioned.

### Hyper-androgenization and hormonal treatment

Female Balb/c mice (Charles River Laboratories Italia s.r.l., Calco, LC, Italy) were maintained on 12-h light, 12-h dark cycles and given food and water *ad libitum*.

All experiments were performed using mice at post-natal day 40 and of weight ranging from 14 to 19 g. In order to hyper-androgenize mice, daily subcutaneous (SC) injection of dehydroepiandrosterone (DHEA; 1.2 mg/mouse/day, derived from 6 mg/100 g body weight [37]) dissolved in 0.1 ml sesame oil were performed for 20 consecutive days. To study the effect of oral administration of recombinant human FSH on PCOS-induced animals, three groups of 8 mice each were used (see the Experimental Design in Table 1). Simultaneously to DHEA treatment, one group (DHEA+rhFSH) received 0.02 IU of rhFSH (Gonal-F, Merck-Serono, Darmstradt, Germany) deriving from the dose of 50 IU in women [17] and recalculated according to the body weight average in the mouse in a total volume of 0.1 ml of saline solution, administred directly into the stomach of mice via oral gavage, once a day for 1 week, with progressive increases of 50 % of initial dose each week for a total of 3 weeks in a step-up approach (Table 1). A second group (DHEA) was treated only with DHEA by SC administration and saline by oral gavage, as previously described. The last group served as controls (CTRL) and only received sesame oil subcutaneously and saline solution by oral gavage throughout the experiment. The body weight of animals was measured at the beginning and at the end of the 20-days treatments.

**Table 1:** Experimental design

Treatments	SC injection	PO administration		
CTRL	Sesame oil	Saline solution		
DHEA	DHEA (1.2 mg)	Saline solution		
DHEA+rhFSH	DHEA (1.2 mg)	rhFSH		
		1^st^ week	2^nd^ week	3^rd^ week
		0.02 IU	0.03 IU	0.04 IU

Daily sub-cutaneous (SC) injection and *per os* (PO) administration for each experimental group composed of 8 mice. The total volume for both the SC injection and the PO administration is 0.1 ml of sesame oil or of saline solution respectively. The treatment continued for 20 consecutive days

### Hormonal analysis of serum

After 20 consecutive days of treatments, blood samples were collected from each mouse before sacrifice. To separate serum, blood was kept at 4 °C for 1 h, followed by two consecutive centrifugations for 10 min at 15000 g at 4 °C. Serum was kept at −20 °C until hormonal assay was performed. Analysis of Testosterone (T) was performed employing a competitive inhibition enzyme immunoassay technique (Mouse Testosterone ELISA Kit, CSB-E05101m, Cusabio, Hubei Province, China; minimum detectable concentration (minDC) = 0.1 ng/ml; intra-assay and inter-assay precisions (mean of the percentages of coefficients of variation) are both <15 %). According to the manufacturer’s instructions, no significant cross-reactivity or interference between mouse testosterone and analogues was observed. Levels of Progesterone (P4), Estradiol (E2) and LH were measured using a magnetic bead immunoassay based on Luminex Multiplex System (Merck Millipore, Darmstadt, Germany), according to the manufacturer’s instructions; respectively E2 and P4 were analyzed by the “Steroid/Thyroid Hormone Magnetic Bead Panel” (# STTHMAG-21K; minDC = 0.02 ng/ml for E2 and 0.09 ng/ml for P4; intra-assay and inter-assay precisions are <10 % for both analytes), while the “Mouse Pituitary Magnetic Bead Panel” (# MPTMAG-49K; minDC = 1.34 pg/ml; intra-assay precision <15 % and inter-assay precision <20 %) was used for LH.

### Morphological evaluation of ovaries

The ovaries were collected immediately after sacrifice and fixed in 10 % neutral buffered formalin (Bio-Optica, Milan, Italy) over night. The ovaries were dehydrated in a graded series of ethanol, cleared with xylene, embedded in paraffin (Bio-Optica) and serially sectioned at 4 μm. Slices were placed on glass microscope slides in traceable order, stained with hematoxylin and eosin (DDK Italia, Vigevano, Italy) and finally analyzed under light microscopy to assess follicles diameter and morphological features [38].

In order to select a representative follicular population to be analyzed in each ovary, we considered one slice every 150 μm (i.e. every 37 sections), resulting in a mean of 4 slices for each ovary. These slices were used to identify the antral follicles to be measured. For each follicle, measurements and morphological evaluation were than conducted on the neighboring slices containing the corresponding equatorial section. The follicle diameter was calculated as the mean distance between opposite basal membrane portions, while the wall thickness was calculated as the sum of theca interna and granulosa cell layers. Averages from three different measurements were considered. The follicular population was divided in two classes according to follicle diameter: 150–300 μm (early-small antral follicles) and >300 μm (large antral follicles) [39–41]. Moreover the presence of morphological cystic signs, as previously described [26, 27, 42–44]), were recorded for each antral follicle. Two investigators performed morphological analysis independently.

### Immunoexpression of aromatase cytochrome P450

Indirect immunohistochemistry was carried out to evaluate the expression and localization of aromatase cytochrome P450 (P450 arom). Before immunohistochemical staining, sections were routinely deparaffinized, rehydrated and successively heated in a microwave oven in 0.01 M citrate buffer. Then sections were incubated with 10 % (v/v) normal rabbit serum, 0.3 % (v/v) Triton X-100 and 3 % (w/v) bovine serum albumin (BSA) in phosphate buffered saline (PBS) for 30 min to block non-specific binding of secondary antibody. Sections were incubated overnight at 4 °C with 4 μg/ml of polyclonal goat anti-CYP19 (CYP19 (C-16): sc-14245, Santa Cruz Biotechnologies, Inc, Dallas, TX, USA;) diluted in PBS with 1 % (w/v) BSA and 0.3 % (v/v) Triton X-100. Primary antibody was detected by using an Alexa Fluor 488-labeled rabbit anti-goat IgG (diluted 1:1000 in PBS with 1 % (w/v) of BSA. Negative controls were performed by omitting the primary antibody. The sections were mounted with an antifade medium Vecta Shield (Vector Laboratories, Inc., Burlingame, CA, USA) supplemented with 1 μg/ml 4′,6-diamidino-2-phenylindole (DAPI). Samples were analyzed on an epifluorescence microscope (Eclipse E600, Nikon Corp., Tokyo, Japan) at a magnification of 200–400×.

### FSH-derived bioactive peptide synthesis

**Fig. 1:**
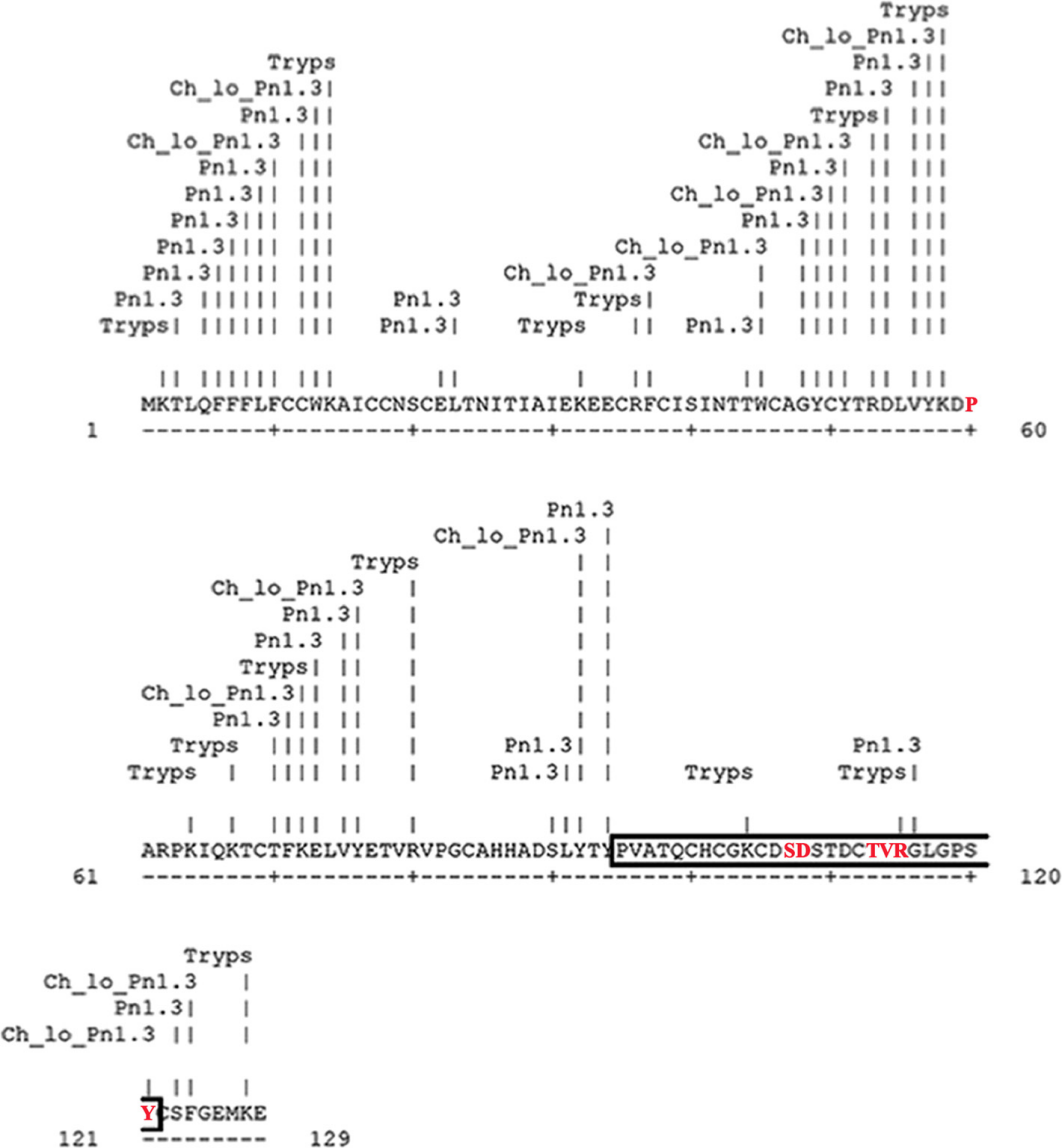
Map of follitropin beta-subunit cleavage sites from stomach and pancreatic proteases digestion: pepsin, trypsin, chymotrypsin. The image derives from resource web portals *web.expasy.org* and databases for protein sequences www.uniprot.org; www.rcsb.org/pdb/). This tool predicts cleavage sites of selected proteases in a protein sequence, in this case pepsin (*Pn1.3*), trypsin (*Tryps*), chymotrypsin (*Ch_Lo*). In the map, the cleavage probability of trypsin and chymotrypsin is 70 % at least. Cleavage occurs at the right side (C-terminal direction) of the marked amino acid. Specific residues for hFSH-receptor binding in the follitropin beta-subunit [48] are identify in *red*. The highlighted peptide sequence composed of amino acids 95–121 likely maintains the biological activity of intact FSH, based on our in silico analysis, and it was used for *in vitro* assay

The FSH sequence ([Uniprot: P01225]; www.uniprot.org/uniprot/P01225; www.rcsb.org/pdb/explore/explore.do?structureId=1XWD) was examined for cleavage sites using Peptide Cutter (web.expasy.org; [45]), a tool that predicts potential digestion sites cleaved by pepsin, trypsin and chymotrypsin. This process resulted in the identification of a peptide sequence of the beta-subunit that likely maintains the biological activity of intact FSH and is composed of amino acids 95–121 (Fig. 1 and Additional file 1: Figure S1). This sequence possesses the amino acid sequence involved in binding to the FSH receptor [46–48]. The identified peptide of 27 amino acids has been chemically synthetized (PRIMM Biotech, Milan, Italy) at a high degree of purity (>95 %) for subsequent biological evaluations.

### Assessment of biological activity of FSH-derived peptide

The activity of the peptide sequence was tested on our standard *in vitro* maturation (IVM) protocol [49]. As biological endpoints, cumulus expansion and meiotic maturation rate were evaluated at the end of IVM period [36].

Bovine ovaries were recovered at the abattoir (INALCA Spa., Ospedaletto Lodigiano, LO, IT 2270M CE, Italy) from pubertal females (4–8 years old) subjected to routine veterinary inspection and in accordance to the specific health requirements stated in Council Directive 89/556/ECC and subsequent modifications. Ovaries were transported to the laboratory within 2 h in sterile saline at 26 °C. All subsequent procedures, unless differently specified, were performed at 35–38 °C and carried out as previously described [50]. Cumulus-oocyte complexes (COCs) were retrieved from mid-sized antral follicles (2–6 mm) with a 16-gauge needle mounted on an aspiration pump (COOK-IVF, Brisbane QLD, Australia) in M199 supplemented with 20 mM HEPES, 1790 units/L heparin and 0.4 % BSA. After examination under a stereomicroscope, only COCs medium-brown in color, with five or more complete layers of cumulus cells enclosing an oocyte with finely granulated homogenous ooplasm were used. Selected COCs were individually cultured for 24 h in M-199 added with 0.68 mM L-glutamine, 25 mM NaHCO_3_, 0.4 % BSA fatty acid free and 0.2 mM sodium pyruvate in humidified air under 5 % CO_2_ at 38.5 °C. The basic culture medium was then supplemented with 10^−1^ IU/ml of rhFSH (as in standard IVM in bovine oocytes [51]) or with the peptide (at the same molar concentration), or not supplemented (control group). A picture for each COC was taken before and at the end of *in vitro* culture and cumulus expansion was calculated as ratio between final cumulus area and initial cumulus area. Cumulus area was measured by ImageJ 1.48v (National Institute of Health, USA; [52]) tools. Oocytes were then mechanically freed from cumulus cells and fixed in 500 μl of 60 % Methanol in Dulbecco’s Phosphate Buffered Saline for 30 min at 4 °C. The oocytes were then stained with 0.5 mg/ml of Propidium Iodide to evaluate meiotic stage by observation at 200–400× under fluorescence microscopy [50]. About 30 COCs were analyzed for each group during three different runs.

### Statistical analysis

Statistical analyses were performed using Prism GraphPad (GraphPad Software, version 6.0f, San Diego, CA, USA). *In vivo* data were analyzed by one-way ANOVA, followed by Fisher’s Least Significant Difference (LSD) multiple comparison test. Fisher’s exact test was used to compare the percentages of atretic/cystic follicles on the total follicle population. Data of cumulus expansion assay were tested for Gaussian distribution using Kolmogorov-Smirnov test. Since these data were not normally distributed, Kruskal-Wallis test, followed by Dunn’s multiple comparison test was used to analyze the cumulus expansion data. Regardless of the test P values <0.05 were considered significant.

## Results

### Body weight increase

**Fig. 2:**
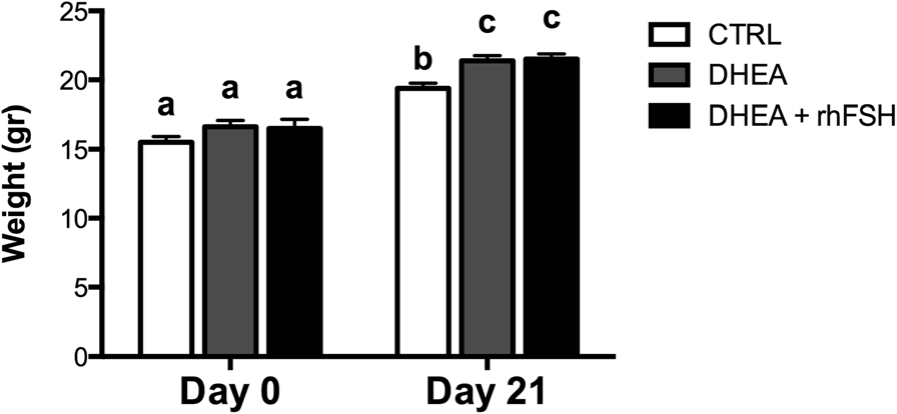
Effect of different hormonal treatments on body weight of mice at day 0 and 21. Data were analyzed by one-way ANOVA, followed by Fisher’s LSD multiple comparison test; different letters indicate significant differences between groups (*P* <0.05)

To investigate the effect of different hormonal treatments on the body mass, mice were weighted during experimentation. Before treatment the body mass of animals was not statistically different between groups (Fig. 2). Mice increased in weight during the treatment phase of this experiment but those treated with DHEA gained approximately 10 % more than controls (Fig. 2).

### Hormonal analysis of serum

**Fig. 3:**
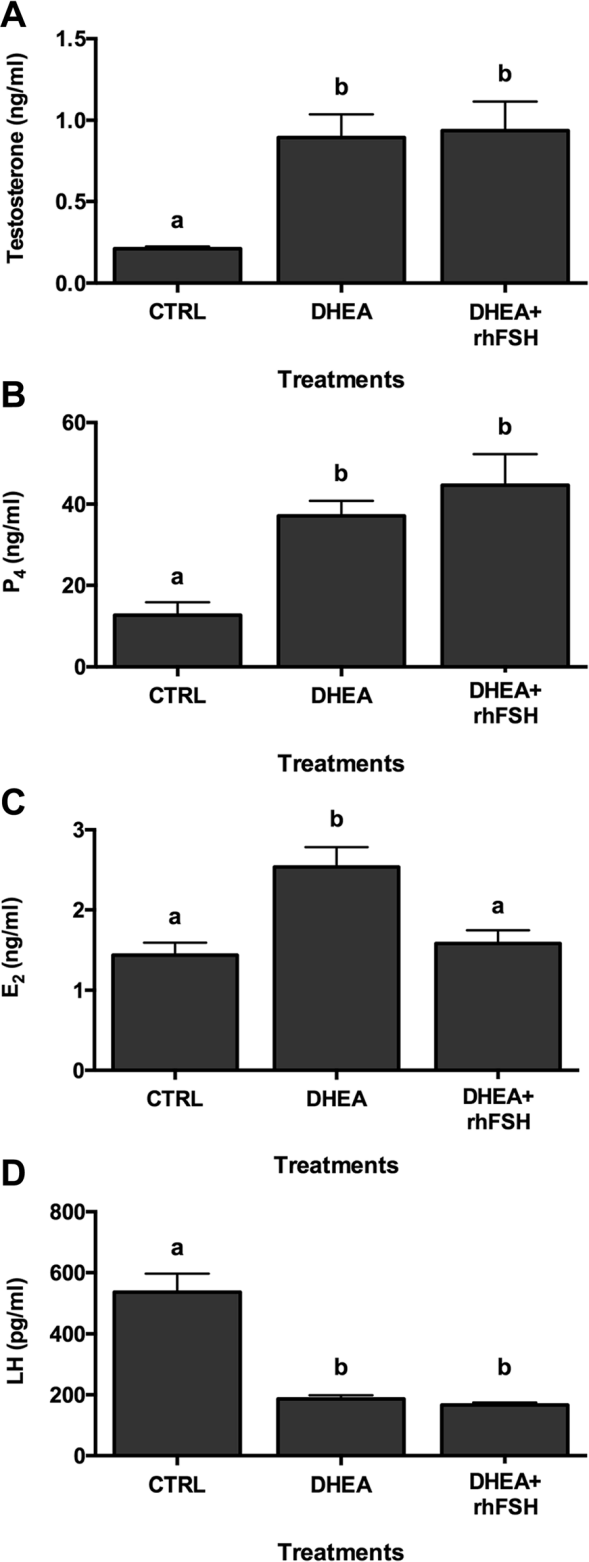
Effect of different hormonal treatments on Testosterone, Progesterone, Estradiol, and Luteinizing Hormone serum concentration. **a** Testosterone; **b** Progesterone; **c** Estradiol; **d** Luteinizing hormone. Data were analyzed by one-way ANOVA, followed by Fisher’s LSD multiple comparison test; in each graph, different letters indicate significant differences between groups (*P* <0.05)

The effect of low dose administration of rhFSH on the serum hormonal profiles was evaluated using immunoassay techniques. Results are illustrated in Fig. 3. Testosterone and P4 concentration were statistically higher in all the PCOS-induced animals (DHEA treated) compared to CTRL, irrespectively to rhFSH administration (Fig. 3a and b respectively). E2 analysis revealed that while DHEA significantly increased serum E2 concentration compared to CTRL, DHEA+rhFSH treatment was able to restore E2 concentration statistically similar to the CTRL (Fig. 3c). Finally DHEA alone or with rhFSH resulted in a statistical decrease of LH concentration respect to CTRL (Fig. 3d).

### Morphological evaluation of ovaries

**Fig. 4:**
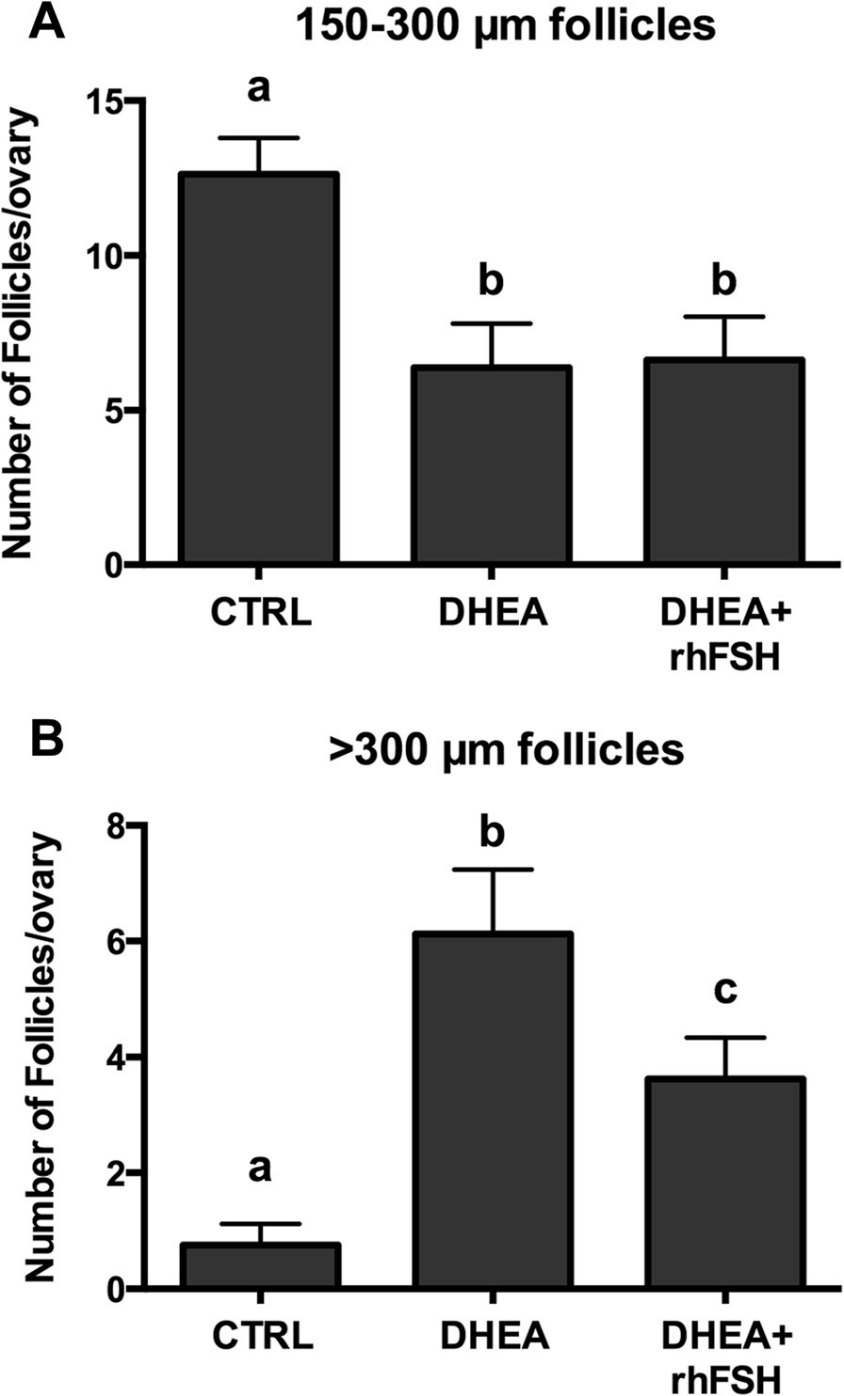
Effect of hormonal treatments on the number of antral follicles per ovary. **a** antral follicles of 150–300 μm in diameter; **b** antral follicles >300 μm in diameter. Data were analyzed by one-way ANOVA, followed by Fisher’s LSD multiple comparison test; in each graph, different letters indicate significant differences between groups (*P* <0.05)

To study the potential role of the oral administration of low doses of rhFSH on follicle development in DHEA-treated animals, the number of follicles 150–300 μm in diameter and of follicles >300 μm in diameter was monitored in each ovary. DHEA statistically reduced the number of small follicles per ovary independently from rhFSH (Fig. 4a). On the other hand, DHEA in the presence or absence of rhFSH importantly increases the number of larger follicles per ovary, but rhFSH attenuated DHEA’s actions, significantly reducing large follicle population (Fig. 4b).

**Fig. 5:**
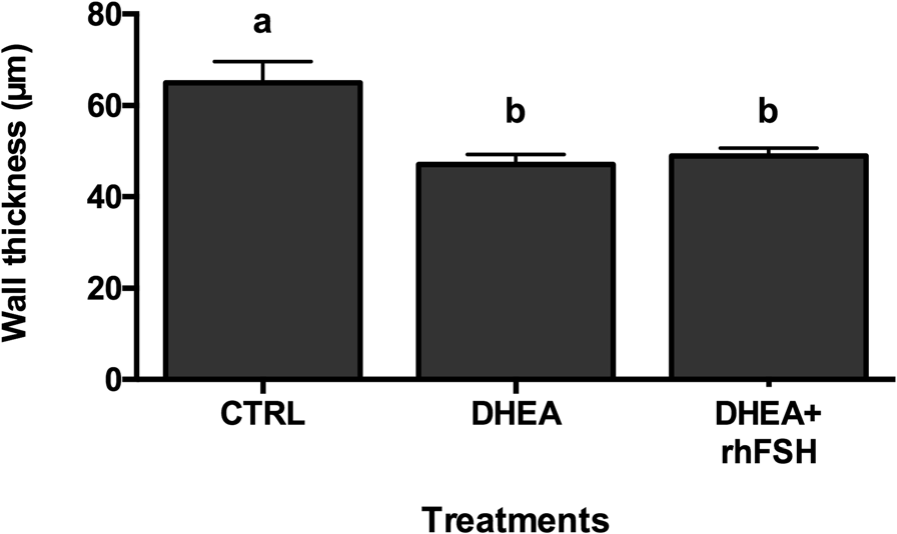
Effect of different hormonal treatments on the wall thickness. The effect was evaluated on the population of antral follicles >300 μm of diameter. Data were analyzed by one-way ANOVA, followed by Fisher’s LSD multiple comparison test; different letters indicate significant differences between groups (*P* <0.05)

The number of granulosa cells as assessed by the thickness of the theca and granulosa cell layers was decreased by DHEA (*P* <0.05), irrespective of rhFSH administration, in the large (>300 μm) follicles (Fig. 5) but not in 150–300 μm follicles (data not shown).

**Fig. 6:**
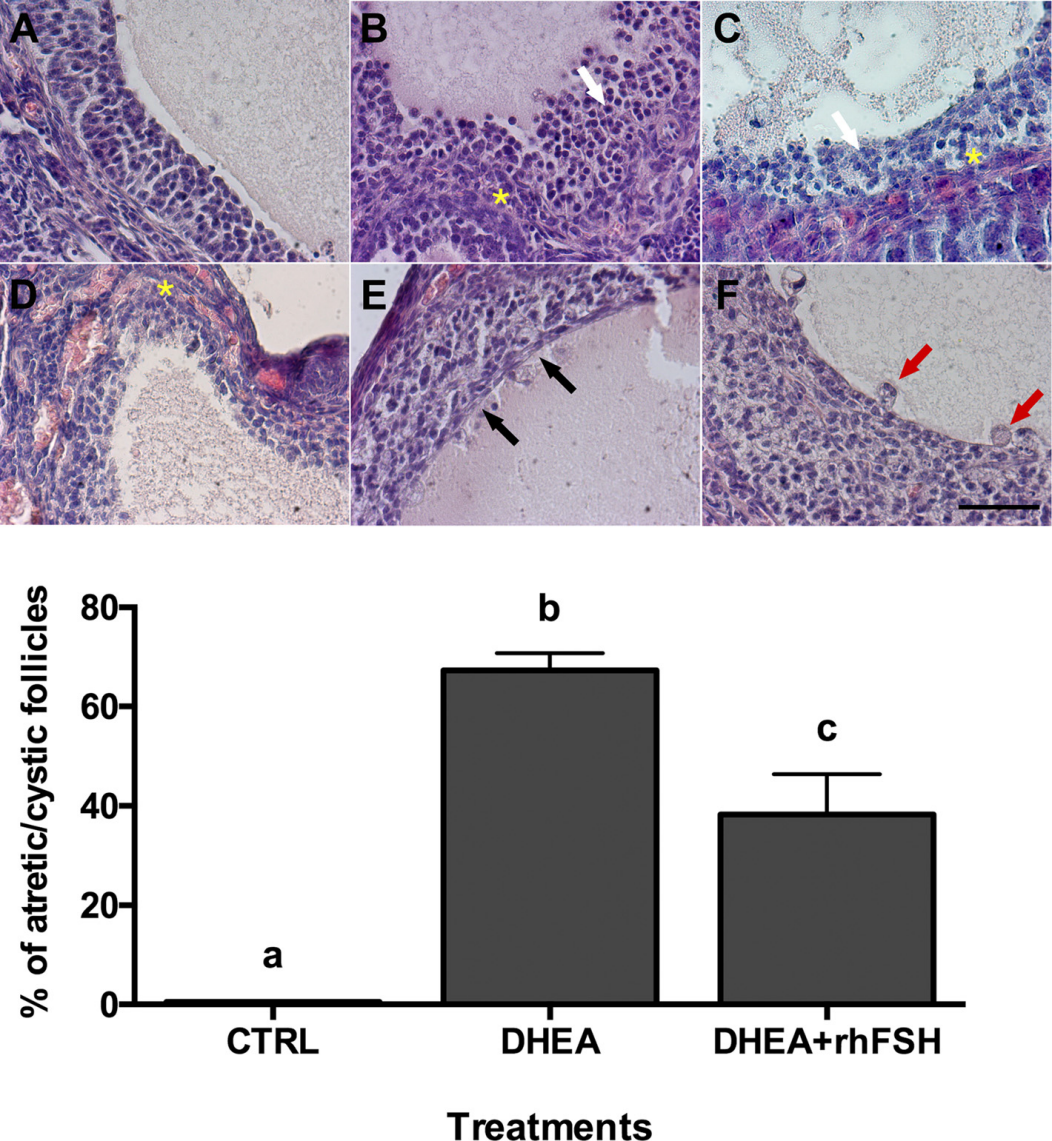
Effect of different hormonal treatments on the percentage of follicles presenting atretic/cystic signs. *Upper panel* Representative images of typical morphological changes in antral follicle walls of ovaries isolated from controls (**a**) compared to DHEA-treated mice (**b**, **c**, **d**, **e**, **f**), stained with hematoxylin and eosin. In the control (**a**) theca externa, theca interna, basal membrane and granulosa cells layers appear normal. **b**, **c** and **d** represent progressive changes associated with follicular atresia (ie. pyknosis, disruption of the basement membrane (*asterisks*) and of granulosa layers (*white arrows*) and invasion of blood cells). Cystic features are described by thin and elongated epithelioid cells in the inner surface of the wall (**e**, *black arrows*) and macrophages in the cystic fluid (**f**, *red arrows*). Bar = 50 μm. *Lower panel* The effect of different hormonal treatment was evaluated on the population of antral follicles >300 μm of diameter. Data were analyzed by one-way ANOVA, followed by Fisher’s LSD multiple comparison test; different letters indicate significant differences between groups (*P* <0.05)

**Fig. 7:**
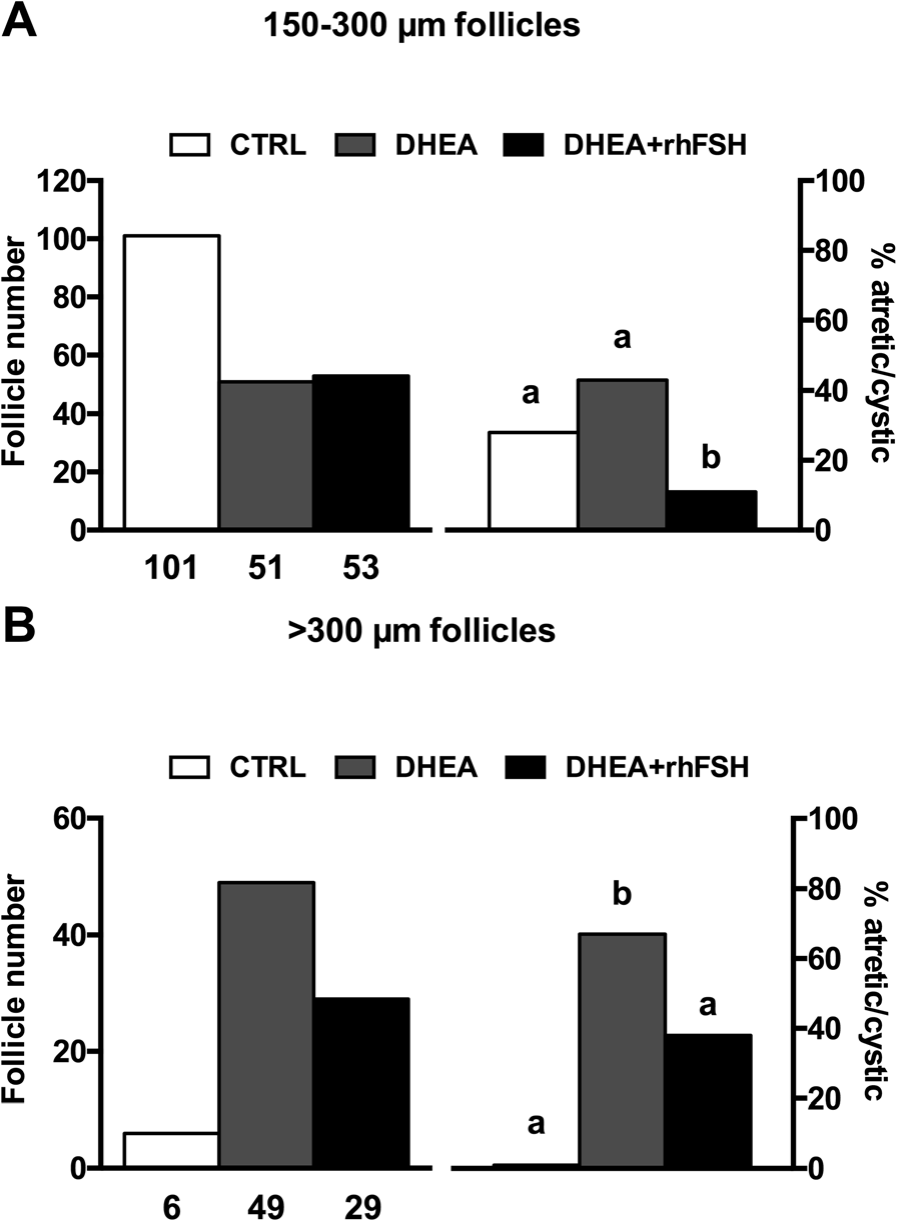
Effect of different treatments on atretic/cystic follicles. **a** Total number (*left*) and percentage of atretic/cystic (*right*) in 150–300 μm follicles. **b** Total number (*left*) and percentage of atretic/cystic (*right*) in >300 μm diameter follicles. Percentages of atretic/cystic follicles were analyzed by Fisher’s exact test; different letters indicate significant differences between groups (*P* <0.05)

Finally, morphological signs of progressive follicle atresia and cystic formations were observed (Fig. 6, upper panel). These include granulosa cell pyknosis (Fig. 6b), disruption of the basement membrane (indicated by asterisks in Fig. 6b, c, d) and granulosa cells layers (pointed out by white arrows in Figs. 6b and c) as well as invasion of blood cells, the presence of elongated epithelioid cells in the inner surface of the follicle wall (indicated by black arrows in Fig. 6e), macrophages in the cystic fluid (showed by red arrows in Fig. 6f), and reduction of granulosa cells layers. DHEA significantly increased the occurrence of morphological cystic features in antral follicles with a diameter >300 μm per ovary (Fig. 6, lower panel); the oral administration of rhFSH in DHEA-treated mice was able to significantly decrease the percentage of atretic/cystic signs in large follicles, even if it is still higher than CTRL (*P* <0.05). Moreover in the 150–300 μm follicle population, rhFSH administration importantly reduced the percentage of atretic/cystic follicles on total follicular population compared to DHEA treatment alone (Fig. 7).

### Localization of aromatase cytochrome P450

**Fig. 8:**
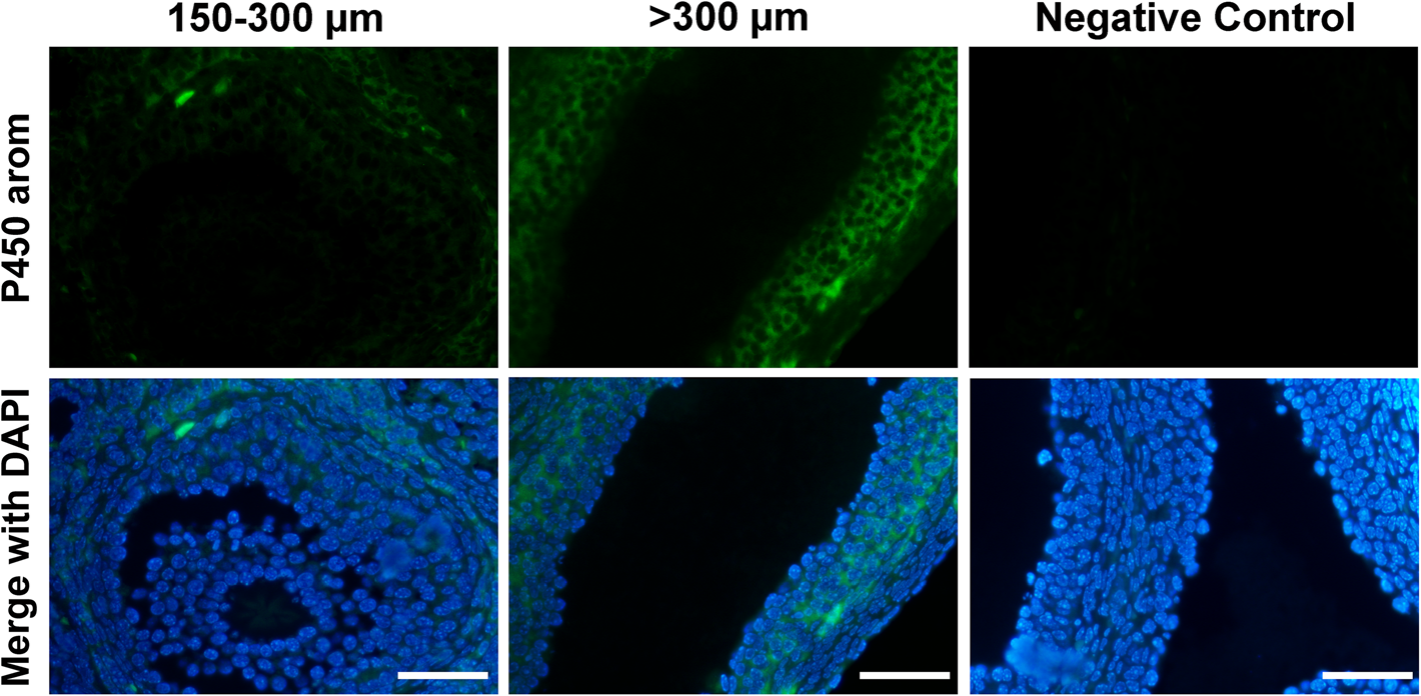
Immunohistochemical localization of aromatase cytochrome P450. Aromatase expression progresses along with follicle development. No or weak aromatase protein was detected in 150–300 μm early-small antral follicles (*left*). Positive staining was evident in >300 large antral follicles and more concentrated inside the cytoplasm of mural cells, lying on the basal membrane (*central*). Control sections did not exhibit any positive staining (*right*). Scale bar = 50 μm

In all ovaries regardless the treatment, P450 aromatase protein was absent or weak in 150–300 μm early-small antral follicles (Fig. 8) while was localized in the cytoplasm of granulosa cells in >300 μm large antral follicles, with P450 arom concentrated inside the cytoplasm of mural cells, lying on the basal membrane (Fig. 8) with little or no staining in the cumulus cells. Control sections did not exhibit any positive staining.

### Cumulus expansion and meiotic maturation rate evaluation

**Fig. 9:**
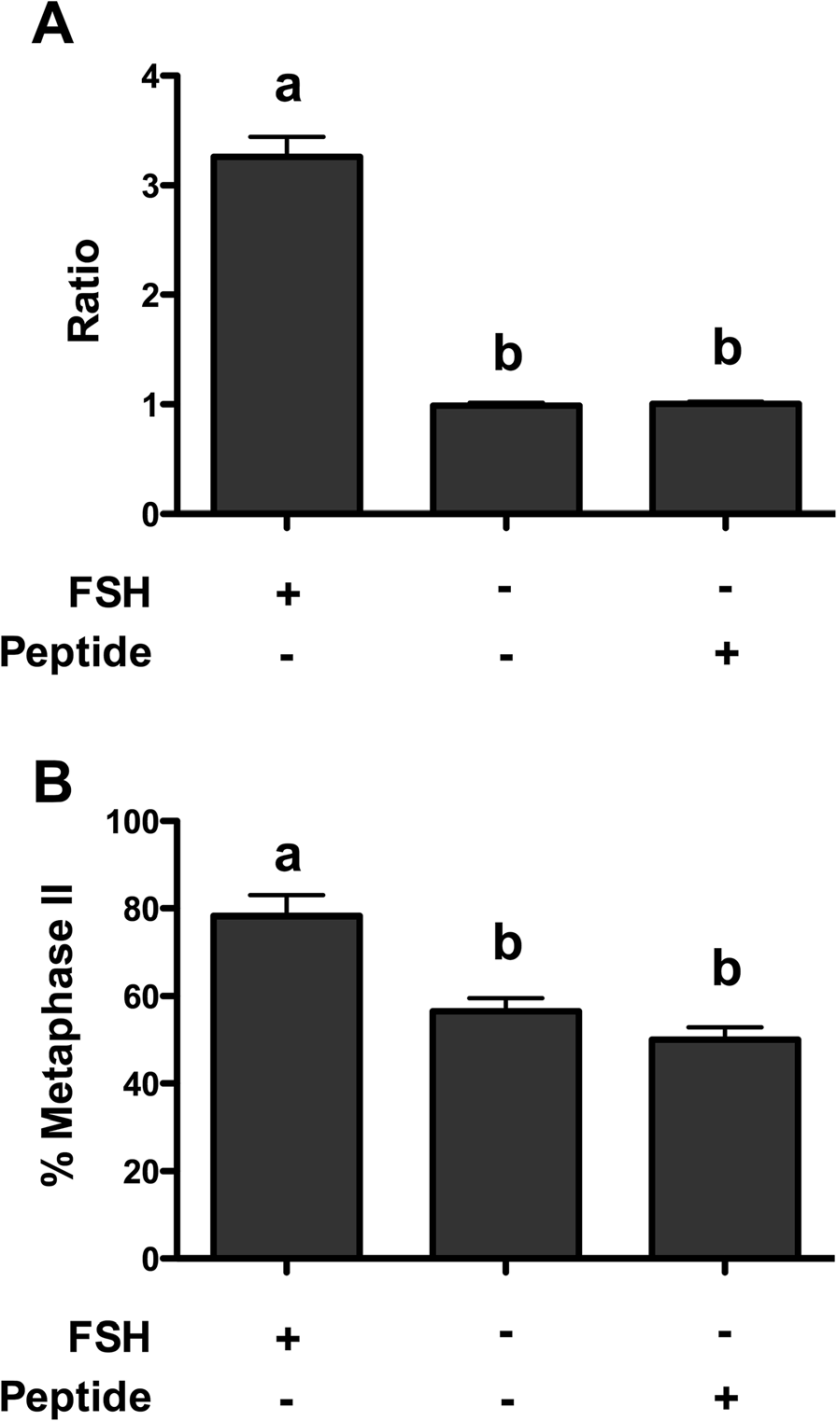
Effect of culture in presence or absence of FSH and in presence of the peptide. **a** The effect was evaluated on ratio between cumulus oophorus area after 24 h culture and at the collection time. Data were analyzed by Kruskal-Wallis test, followed by Dunn’s multiple comparison test; different letters indicate significant differences between groups (*P* <0.05). **b** The effect was evaluated on oocyte maturation rate, as the ability to reach metaphase II stage of meiosis. Data were analyzed by one-way ANOVA, followed by Fisher’s LSD multiple comparison test; different letters indicate significant differences between groups (*P* <0.05)

The biological effect of the peptide derived from in silico enzymatic digestion of FSH was analyzed on the cumulus expansion and on the maturation rate of bovine COCs. The peptide did not induce cumulus expansion (Fig. 9a), since the ratio between cumulus areas after culture and before culture was similar to that of COCs cultured in absence of FSH (*P* >0.05). Moreover the FSH peptide was not able to promote oocyte maturation (Fig. 9b), even when used at concentration 100, 1000 or 10000 times higher (data not shown). Moreover, treatment with the peptide concurrently to FSH did not affect the biological effects, since cumulus expansion index and maturation rate were comparable to that of FSH alone (data not shown).

## Discussion

Most of the animal models used to understand the development of PCOS-related dysfunctions are based on induced hyperandrogenism [53]. DHEA, an androgen of mainly adrenal origin, is often increased in women with PCOS [54]. Therefore DHEA is utilized to induce PCOS in different rodent models. The dose commonly used (6 mg/100 g body weight) ensures a hyperandrogenized status equivalent to that found in women with PCOS [55]. The treatment that we utilized is commonly used in mice to induce the PCOS phenotype, characterized by infertility and ovaries containing more atretic follicles and follicular cysts [56–58]. Our data confirm the previous finding that DHEA treatment increased the number of large follicles (>300 μm of diameter) per ovary, simultaneously reducing the small antral follicles population. In addition to the increase in follicles number, our results demonstrated that DHEA treatment induced an increase of the number of large follicles presenting morphological signs of atresia and/or cysts (from 0.0 % in the Control group to 67.26 % ± 3.5 in the DHEA group). Motta and colleagues found that mouse cysts had a thin theca layer and a packed stratum of granulosa cells [55]. These findings are confirmed by our results, since DHEA treated mice exhibit a reduced thickness of follicular wall (theca interna layer plus granulosa cell layer) in the large follicles. We observed also that both serum E2 and P4 levels were increased with induction of cysts formation. These findings are in agreement with other authors [26, 28, 59, 60] who observed increase in both cytochrome P450 17-hydroxylase and steroidogenic acute regulatory protein (StAR) activities in theca cells from women with PCOS, suggesting a global enhancement of steroidogenesis. This is also consistent with studies on cultures of human theca cells derived from follicles isolated from the ovaries of PCOS and normal women where it has been demonstrated that PCOS theca cells produce greater amounts of testosterone, 17-hydroxyprogesterone and P4 than normal theca cells [61]. Although the mechanism for LH hypersecretion described in human PCOS is not entirely clear, some data suggest that it involves impaired negative feedback on LH secretion mediated by either high E2 or P4 levels in women with PCOS [62]. Unexpectedly in our study, serum LH was decreased while LH over-production is commonly considered one of the peculiar trait of PCOS [63]. While in the rat data on the effect of postnatal treatment with DHEA on LH concentration are reported, although contrasting [28, 29, 64], to the best of our knowledge this is the first study showing the effect on LH concentration after 20 days of DHEA administration in the mouse model.

Moreover, our results reveal that DHEA increased animal weight at the end of the treatment. These observations are in agreement with both the wide distribution of obesity in PCOS-affected women [65] and with the characterization of the murine model reported in literature [66].

The therapy of PCOS is focused on ovulation induction in those desiring pregnancy. This may be achieved indirectly with clomiphene citrate (CC), which is an oral selective estrogen receptor modulator which action results in increases in circulating FSH [13], or directly by FSH administration. The first line of treatment, CC, restores ovulation in about 80 %, but will result in pregnancy in only 35 % of patients [67]. Additionally, about 25 % of PCOS women do not respond to CC and are considered to be “clomiphene resistant” [67]. Even though FSH treatment is more effective than CC, it is still considered a second line of treatment, mainly because of the lack of oral preparation and the risk of adverse effects [13, 14]. In order to reduce the risk of OHSS and multiple pregnancy, low-dose treatment programs have been successfully implemented [17, 18, 20]. The basic thinking behind this regiment is the “threshold theory”, which demands the attainment and maintenance of follicular development with exogenous FSH without exceeding the threshold requirement of the ovary [67]. A low starting dose of FSH (usually 50–75 IU) is used for 7–14 days and, if necessary, a weekly 50 % increment of the initial or previous amount is administered until follicular development is initiated [17].

In our study we investigated the possibility of FSH oral administration. Gonadotropins, as most of the drugs constituted by protein formulations, are traditionally delivered via intramuscular, subcutaneous, or intravenous routes because of their reduced oral bioavailability. This is mainly due to the fact that peptides can be readily degraded and pass poorly through the intestinal mucosa [68, 69]. On the other hand, to the best of our knowledge this is the first study reporting that an oral administration of a low dose of rhFSH is able to ameliorate some peculiar features of PCOS in hyper-androgenized mice with treatment using higher doses potentially being more effective. However, this remains to be determined.

During the first week, we orally administered 0.02 IU/die of rhFSH, which was derived from the dose of 50 IU in women and recalculated according to the average body weight in the mouse. The orally administered rhFSH was able both to reduce the number of large antral follicles and at the same time to maintain their viability, while reducing the percentage of atretic and cystic signs. The effect of FSH administration on cystic signs in ovarian follicles has not been extensively investigated, apart from a study in a Guinea pig model, which demonstrated that exogenous FSH treatment effectively reduced the ovarian cyst formation [70]. In our PCOS model, the oral administration of rhFSH normalized estradiol serum concentration. This phenomenon appears to reflect the follicular distribution after the oral administration of rhFSH, since rhFSH treated ovaries have a decrease in large antral follicles number (Fig. 4b) in favor of an increase in the new population of small growing healthy follicles (Fig. 7a). Compared to larger follicles, these smaller growing follicles synthetize and secrete less E2 than the larger follicles [71, 72], and this could account for the reduced E2 in the rhFSH treated mice. Further support from this concept is provided by the immunohistochemical detection of P450 aromatase in >300 μm large antral follicle and not in the smaller follicles. Thus the observed 50 % decrease in serum E2 observed in the rhFSH treated ovaries directly corresponds the 50 % decrease in the number of aromatase expressing follicles.

Thus rhFSH, even though orally administered, seemed to be able to partially restore the physiological status of ovarian follicles considering both morphological and functional aspects. During estrous cycle, FSH stimulates proliferation of granulosa cells in primary follicles, and once a follicle reaches a diameter of approximately 150 μm, FSH induces antrum formation and aromatase activity within the granulosa cells with a gradual increase in estradiol synthesis [73, 74]. Moreover, our results demonstrate the feasibility of administering effective gonadotropin treatment orally.

However while oral administration is effective, we hypothesize that intact FSH is unlikely to be transferred from the gastrointestinal tract into the circulation, even though we cannot exclude it. In the present study we tested whether a bioactive peptide derived from FSH is functionally active. From this premise, we selected an amino acidic sequence derived from the FSH molecule digestion and containing amino acids responsible for specific receptor binding. Our results indicated that the selected peptide (from the amino acid 95–121 of β-chain) is not able to exert biological effects (i.e. cumulus expansion and oocyte maturation) in an *in vitro* model in the bovine species.

On the other hand World Health Organization has calculated that over 10 % of women are inflicted by infertility and subfertility (www.who.int). Assisted reproductive technologies (ART) are well-established treatments, representing substantial economic and healthcare implications for patients. Total number of ART cycle per annum will reach 2 million by the end of 2015 with a forecast cost for therapeutics market that is estimated in about 8 billion of euros. This valuation derived from the mean of direct cost of one fresh ART treatment cycle in several countries [75]. Considering that one treatment cycle is often not enough to achieve the childbirth, the amount is surely underestimated. This framework includes also the therapeutic market linked to PCOS considering also that the pharmaceutical market is evolving in a context of increasing economic pressure, it demands alternative approaches [76]. This situation has contributed to a revival of interest in peptides as potential drug candidates, considering alternative routes of administration. The peptide drug market is also growing twice as fast in the worldwide drug market [77]. Certainly peptides and proteins offer several advantages as compared to conventional drugs. These include high activity, high specificity, low toxicity, and minimal non-specific and drug-drug interactions [22], but the physiological, enzymatic and chemical barriers for oral administration route pose a significant challenge to the delivery of peptide and protein drugs [76]. Then oral delivery of peptides and proteins, and in particular of FSH [78], is currently a topic of intense research, which, together to the economic implication of ovarian stimulation not only in PCOS treatment [22, 78], strongly encourages further investigation in identifying FSH-derived peptides or a combination of peptides that have biologically activity.

## Conclusions

In summary, DHEA treatment induced a global increase of Testosterone, E2 and P4 level confirming data from previous studies on DHEA-induced PCOS model in mouse where postnatal treatment of mice with DHEA for 20 consecutive days resulted in most females exhibiting follicular cysts with a thin granulosa cell layer and anovulation, increased numbers of atretic follicles, hyperandrogenism, and altered ovarian steroidogenesis with elevated serum levels of androgens, estrogens and progesterone [26, 55–57, 66, 79, 80]. Several evidences indicate that androgens modulate follicle development from the present and from other studies [81–83]. In particular, androgen-receptor KO mouse models have been used to establish that androgens actions through androgen-receptors are actually necessary for normal ovarian function and female fertility and recently has been demonstrated that androgens regulate ovarian follicular development by increasing follicle stimulating hormone receptors [83]. In the present study FSH treatment restored E2 to the control level and we hypothesize that this maybe due to FSH ability to suppress growth of large follicles >300, where aromatase is more expressed. However, how FSH affects DHEA-mediated follicle development in mouse model is unknown and to the best of our knowledge this is the first study showing FSH capability to modulate its activity. The mechanism involved in this interaction deserves certainly to be the subject of future research.

Finally, our results suggest that oral administration of FSH is able to attenuate some of the characteristic of PCOS in a hyperandrogenized mouse model. Although further investigation in identifying FSH-derived peptides potentially maintaining its proper biological activity are needed, this research could improve our understanding of the pathogenesis as well as lead to the development of innovative, more comfortable and cost effective treatments for PCOS infertility.

## Additional file

Additional file 1:
**CLUSTAL multiple sequence alignment scheme of β-FSH. Sequence matching of β-subunits of FSH in bovine, murine and human species and in comparison to the peptide sequence utilized in the** ***in vitro*** **assay.** Asterisks indicate positions that have a fully conserved residue. (Clustal Omega: http://www.ebi.ac.uk/Tools/msa/clustalo/). Compared to the human sequence chosen for the peptide *in vitro* study, this segment in bovine FSH contains two different amino-acids. Neither of these amino acids residues affects the specific residues for hFSH-receptor binding as identified in literature [48]. (TIFF 393 kb)
