# Supplementary figures and images for: Effect of oral administration of low-dose follicle stimulating hormone on hyperandrogenized mice as a model of polycystic ovary syndrome
Source: J Ovarian Res. 2015 Oct 6;8:64. doi: 10.1186/s13048-015-0192-9 (PMC4594749; doi:10.1186/s13048-015-0192-9)

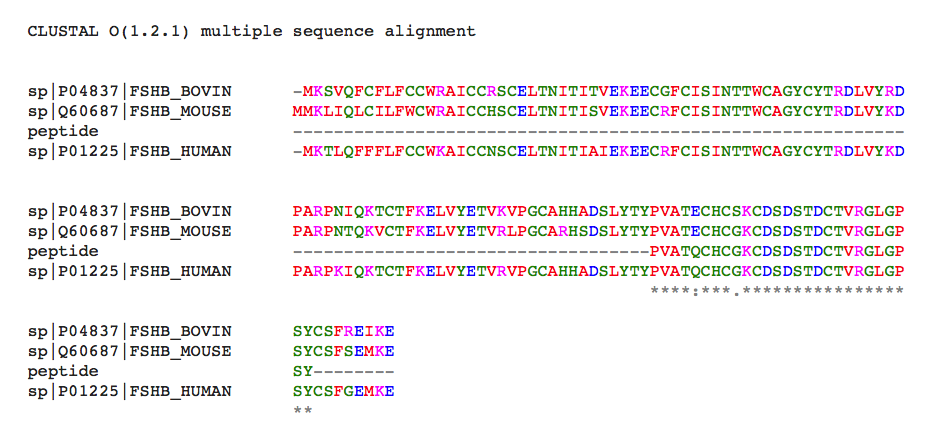

Supplement: Additional file 1: — CLUSTAL multiple sequence alignment scheme of β-FSH. Sequence matching of β-subunits of FSH in bovine, murine and human species and in comparison to the peptide sequence utilized in the in vitro assay. Asterisks indicate positions that have a fully conserved residue. (Clustal Omega: http://www.ebi.ac.uk/Tools/msa/clustalo/). Compared to the human sequence chosen for the peptide in vitro study, this segment in bovine FSH contains two different amino-acids. Neither of these amino acids residues affects the specific residues for hFSH-receptor binding as identified in literature [48]. (TIFF 393 kb) [file 13048_2015_192_MOESM1_ESM.tif]
